# Integrated analysis of risk factors, visual prognosis, and pathogens in pediatric post-traumatic endophthalmitis: a retrospective cohort study

**DOI:** 10.3389/fmed.2026.1699901

**Published:** 2026-01-22

**Authors:** Huanjun Kang, Yifan Wang, Liuqing Xin, Jinchen Jia, Yiming Fan, Shaolei Han, Fang Liu, Suige Qi, Suhuan Sun, Zhiqiang Yue, Tao Huo, Jingxuan Xu, Shanyu Li, Yinbo Zhang

**Affiliations:** Hebei Key Laboratory of Ophthalmology, Hebei Eye Hospital, Hebei Provincial Clinical Research Center for Eye Diseases, Xingtai, Hebei, China

**Keywords:** inflammatory markers, pathogen analysis, pediatric infectious endophthalmitis, pediatric ocular trauma, risk factors

## Abstract

**Aim:**

Research on pediatric ocular trauma remains limited, and clinical management is often extrapolated from adult data. This study aimed to analyze the risk factors, visual prognosis, and microbiological characteristics of infectious endophthalmitis following pediatric ocular trauma, thereby providing evidence for clinical decision-making.

**Methods:**

A retrospective cohort study was conducted on 108 hospitalized children with ocular trauma treated at Hebei Eye Hospital between January 2019 and June 2025. Three parallel analyses were performed within the same population: (1) 54 children (54 eyes) with post-traumatic infectious endophthalmitis (endophthalmitis group) were matched to 54 children (54 eyes) without endophthalmitis (control group). Clinical features and inflammatory markers were compared, and risk factors were identified using receiver operating characteristic (ROC) curves and logistic regression; (2) patients were categorized into good-vision (55 cases) and poor-vision (33 cases) groups. Baseline data and inflammatory indices were compared to classify independent risk factors for poor visual outcome; and (3) pathogen culture and antibiotic susceptibility results were summarized.

**Results:**

A total of 108 children were included, of whom 83 (76.85%) were male and 98 (90.74%) were rural residents. Risk factors for post-traumatic infectious endophthalmitis included delayed presentation, elevated white blood cell count (WBC), neutrophils (NEUT), monocytes (MON), monocyte-to-lymphocyte ratio (MLR), systemic inflammation response index (SIRI), and systemic immune-inflammation index (SII) (OR = 0.979, 1.413, 1.29, 51.404, 166.58, 2.019, and 1.001, respectively). WBC (OR = 1.404) was identified as an independent risk factor. WBC presented good diagnostic performance with an AUC of 0.722; combined predictors improved the AUC to 0.745. Risk factors for poor visual prognosis included endophthalmitis, lens injury, elevated WBC, and elevated NEUT (OR = 4.667, 6.176, 1.152, and 1.15, respectively), with a combined AUC of 0.732. Among culture-positive cases in the endophthalmitis group, bacterial infection predominated (81.48%, 22/27). Gram-positive cocci were most common, primarily *Staphylococcus* and *Streptococcus* species, with broad susceptibility to cephalosporins, penicillin, vancomycin, aminoglycosides, and fluoroquinolones.

**Conclusion:**

Pediatric traumatic infectious endophthalmitis predominantly occurs in male children from rural areas. WBC is a valuable diagnostic biomarker, and the combination of multiple inflammatory indices further improves diagnostic accuracy. Delayed medical consultation is a critical risk factor. Patients with less severe lens damage and lower inflammatory marker levels are more likely to achieve favorable visual outcomes. Bacterial infections, especially Gram-positive cocci, are the predominant pathogens.

## 1 Introduction

Ocular trauma is one of the primary causes of severe visual loss in children. Due to immature behavioral control, children are more vulnerable to injuries than adults. Moreover, their limited ability to cooperate with examinations makes pediatric ocular trauma assessment more challenging, often leading to misdiagnosis or delayed treatment, thereby increasing the risk of endophthalmitis (1–3). In various studies on post-traumatic endophthalmitis, the proportion of children ranges from 2.8 to 71.8%, with a relatively higher incidence in developing countries and poor visual outcomes (4–7). Infective endophthalmitis is one of the most serious and devastating complications of ocular trauma, and early diagnosis and treatment are important to maintain ocular integrity and improve the chance of visual recovery.

Despite recent advances, the overall prognosis remains poor, and only a few studies have specifically investigated traumatic pediatric infectious endophthalmitis. Traumatic pediatric infectious endophthalmitis can lead to severe long-term visual impairment, thereby negatively affecting children’s quality of life, educational opportunities, and socioeconomic prospects, and in addition, the disease places a heavy burden on families and healthcare systems (8). Consequently, this study retrospectively investigated the clinical features, inflammatory parameters, and pathogenic microorganisms of pediatric ocular trauma patients in Hebei Eye Hospital to identify key risk factors, evaluate potential inflammatory biomarkers, and correlate these findings with patient outcomes, thereby guiding future management and improving prognostic accuracy for pediatric patients with post-traumatic infectious endophthalmitis.

## 2 Materials and methods

### 2.1 General information

A total of 108 pediatric patients with ocular trauma, including 83 male and 25 female patients, were retrospectively analyzed after hospitalization at Hebei Eye Hospital between January 2019 and June 2025. Among them, 54 children developed infectious endophthalmitis (endophthalmitis group), while 54 children without endophthalmitis served as the control group. This study was reviewed and approved by the Ethics Committee of Hebei Eye Hospital (No. 2025LW07), and the procedures were conducted in accordance with the Declaration of Helsinki. Owing to the retrospective design, the requirement for informed consent was waived.

### 2.2 Grouping of the patients

The criteria for inclusion in the infectious endophthalmitis group included: (1) confirmed diagnosis of endophthalmitis: patients presented with ocular pain, vision loss, and redness, accompanied by typical clinical signs of infectious endophthalmitis, such as hypopyon and vitreous opacities on slit-lamp examination, or inflammatory changes detected by ocular ultrasonography and other ancillary tests; (2) evidence of infection: pathogens such as bacteria and fungi were identified by smear, intraocular fluid culture, or pathogenic nucleic acids detected by molecular biology methods to confirm the cause of infection; and (3) complete medical records: detailed medical history, ophthalmic examination results, treatment process, and follow-up data were available to comprehensively analyze and evaluate the cases.

**Figure 1:**
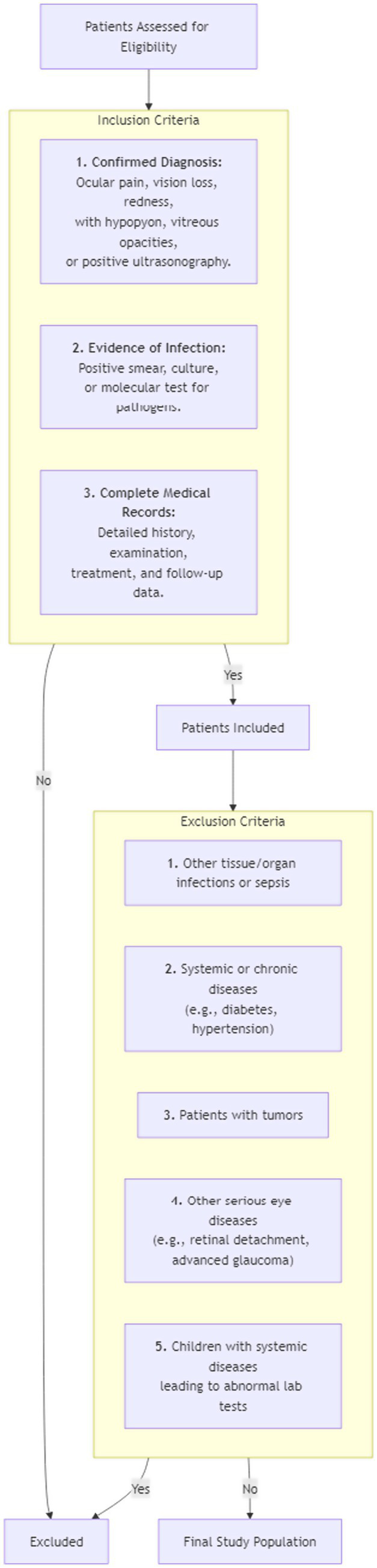
The inclusion and exclusion process of this study.

The exclusion criteria were as follows: (1) patients with other tissue and organ infections in addition to eye infections, or diagnosed with sepsis; (2) patients with systemic diseases or chronic diseases, such as diabetes, hypertension, coronary heart disease, and thyroid disease; (3) tumor patients; (4) history of other serious eye diseases, such as retinal detachment and advanced glaucoma, which may interfere with the diagnosis and treatment of infectious endophthalmitis or affect the efficacy evaluation; and (5) children suffering from systemic diseases such as viral fevers and post-immunization reactions, or other diseases that may lead to abnormal laboratory tests. The flowchart of inclusion and exclusion criteria is shown in Figure 1.

Patients were divided into two groups according to whether infectious endophthalmitis occurred after pediatric ocular trauma: those who met the infectious endophthalmitis inclusion and exclusion criteria after pediatric ocular trauma were included in the endophthalmitis group, and patients who did not develop endophthalmitis after ocular trauma in the same period were selected as the control group.

Prognosis grouping: A favorable visual outcome was defined as a final best-corrected visual acuity (BCVA) ≥ 20/200, whereas BCVA < 20/200 was considered a poor prognosis (7).

### 2.3 Data acquisition

Data collected included sex, age, laterality of injury, family background, injury zone, time from trauma to presentation, causative object/mechanism, first laboratory test results within 24 h after admission, surface of the wound or vitreous or aqueous humor culture results, presenting and discharge visual acuity, and imaging findings. Venous blood samples were collected from all ocular traumatic patients within 24 h after admission. Prior to collection, patients fasted for at least 8 h and avoided strenuous exercise. WBC, NEUT, Lymphocytes (LYM), Monocytes (MON), and Platelets (PLT) were collected in disposable vacutainer tubes containing EDTA-K2 agent. Serum was separated within 2 h after venous blood collection using separation gel vacuum tubes containing coagulants. Samples that could not be analyzed immediately were stored at 4 °C and analyzed within 24 hours. When the wound involved the cornea or corneoscleral limbus and disrupts the integrity of the anterior chamber, aqueous humor was preferentially aspirated as a sample to assess the risk of anterior segment contamination. If the wound was a full-thickness posterior scleral laceration with vitreous prolapse, the prolapsed or surgically obtained vitreous was directly collected as the sample, as it provided a more accurate representation of the infectious status of the posterior segment and the entire globe. In case of high clinical suspicion of infectious endophthalmitis necessitating emergency surgery, vitreous fluid was aspirated under sterile conditions and sent for analysis. For anaerobic culture, blood agar and MacConkey agar were employed as differential media. Specimen testing commenced only after verifying instrument functionality, confirming that quality control was within acceptable limits, and resolving potential interfering factors such as lipemia or specimen agglutination. Specimens with partial defects from incomplete Gram staining were designated with “#.” All laboratory test results were obtained prior to treatment and analyzed in the same laboratory, with reference ranges kept consistent.

A panel of hematological indices—specifically, the neutrophil-to-lymphocyte ratio (NLR), derived neutrophil-to-lymphocyte ratio (dNLR), monocyte-to-lymphocyte ratio (MLR), neutrophil-monocyte-to-lymphocyte ratio (NMLR), systemic inflammatory response index (SIRI), and systemic immune-inflammatory index (SII)—was calculated from routine blood parameters and used to assess patients’ immune status. The formulas were defined as follows: NLR = neutrophil count/lymphocyte count; dNLR = neutrophil count/(leukocyte count-lymphocyte count); MLR = monocyte count/lymphocyte count; NMLR = (monocyte count + neutrophil count)/lymphocyte count; SIRI = neutrophil count × monocyte count/lymphocyte count; and SII = platelet count × neutrophil count/lymphocyte count.

### 2.4 Management strategy

After admission, slit-lamp examination and orbital CT were performed to detect intraocular or orbital foreign bodies. For patients with mild vitreous opacity, emergency primary wound closure combined with intravitreal injections were performed, and part of the aqueous humor and vitreous were drawn and sent for bacterial culture and drug sensitivity test. Intravitreal injections of 0.1 mL norvancomycin hydrochloride at a concentration of 1 mg/0.1 mL and ceftazidime at a concentration of 2.25 mg/0.1 mL were administered to observe the changes in the postoperative condition. If the patient’s symptoms worsened the next day, emergency vitrectomy was performed. For patients with hypopyon or obvious vitreous opacity or intraocular foreign body retention, or retinal detachment, emergency vitrectomy was performed. Vitreous was drawn before vitrectomy and sent for bacterial culture and drug sensitivity test. Perfusion fluid, air, inert gas, or silicone oil tamponade was used according to intraocular tissue injury during surgery. In those who underwent silicone oil tamponade, segmental resection of the inferior iris root was performed. Vitrectomy was performed with 25G vitrectomy. After surgery, broad-spectrum antibiotics were applied systemically and locally.

### 2.5 Statistical analysis

Normally distributed data are presented as mean ± standard deviation (x ± s), and skewed distribution data are presented as median and quartiles M (P25, P75). SPSS 26.0 statistical software was used for data analysis, an independent sample t-test was used for normal data, and the Mann–Whitney U test was used for comparison between two groups for non-normal data. The chi-square test was used for enumeration data and grade data. Factors that were statistically significant were subjected to univariate logistic regression, and factors with a *p*-value of < 0.05 on univariate regression analysis were included in the multivariate logistic regression model. Receiver operating characteristic (ROC) curves were plotted, area under curve (AUC) was calculated, and the optimal cut-off value corresponding to the maximum Youden index value was selected to evaluate the diagnostic efficacy of each inflammatory indicator. Differences were considered statistically significant at a *p*-value of < 0.05.

## 3 Results

### 3.1 Clinical characteristics of the pediatric post-traumatic infectious endophthalmitis group versus the control group

#### 3.1.1 Baseline characteristics

A total of 108 children were included in this study, including 83 male (76.85%) and 25 female patients (23.15%), with a mean age of 7.23 ± 3.88 years and an age range of 1–18 years, of which 10 were urban residents (9.26%) and 98 were rural residents (90.74%). Patients were divided into the infectious endophthalmitis group and the control group based on whether infectious endophthalmitis developed following pediatric ocular trauma. The clinical data of the two groups are presented in Table 1. Statistical differences were observed in family background, treatment time, and visual acuity after treatment between the two groups (*p* < 0.05).

**Table 1:** Clinical characteristics of the pediatric infectious endophthalmitis group and the control group.

Factor	Infected endophthalmitis group (54 eyes)	Control group (54 eyes)	*p*-value
Sex, M/F	41/13	42/12	0.130
Eye, Right/Left	23/31	25/29	0.318
Family background, rural/urban	50/4	48/6	0.037*
Age, years	7 (4, 9)	7 (4, 11)	0.081
Injury-to-presentation time, hours	25 (6.75, 96)	6 (3, 22.25)	0.000*
Post-treatment visual acuity #, ≥0.1 < 0.1	20/24	35/9	0.001*
Gram stain #, G+/G−	23/4	12/1	0.898
Pars plana vitrectomy, Yes/No	52/2	5/49	0.000*

A comparison between the infectious endophthalmitis group and the control group revealed statistically significant differences in family background, injury-to-presentation time, and post-treatment visual acuity, Variables such as gender, eye, family background, post-treatment visual acuity, Gram staining results, pars plana vitrectomy were analyzed using the chi-squared test, whereas age and injury-to-presentation time were assessed using the independent samples Mann–Whitney *U*-test (*p* < 0.05).

#### 3.1.2 Causes of ocular trauma

**Figure 2:**
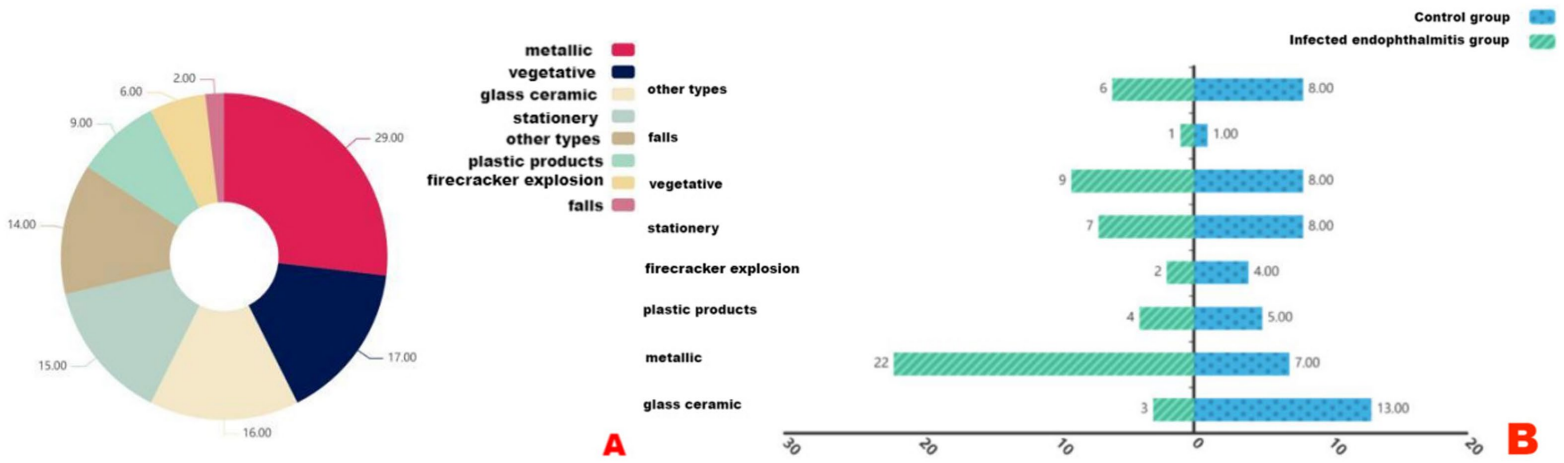
**(A)** The etiological classification of trauma and **(B)** the causes of infectious endophthalmitis group and control group, respectively.

According to statistical analysis, the causes of ocular trauma were classified into eight categories: metallic, vegetative, glass/ceramic, stationery, other types, plastic products, firecracker explosion, and falls. However, the causes of ocular trauma were slightly different between the two groups. In the endophthalmitis groups, metallic injuries accounted for the largest proposition (40.74%, 22/54), whereas, in the control group, glass/ceramic injuries were most common (24.07%, 13/54) (Figure 2).

#### 3.1.3 Inflammatory markers

##### 3.1.3.1 Comparison of inflammatory markers

In this retrospective study, routine blood tests were routinely performed in all children before surgery, so there were no missing values in the data. The distribution of inflammatory markers in the endophthalmitis group and the control group is shown in Table 2. According to the Mann–Whitney test, WBC, NEUT, MON, MLR, SIRI, and SII in the endophthalmitis group were higher than those in the control group, and the differences were statistically significant (*p* < 0.05).

**Table 2:** Inflammatory markers of the pediatric infectious endophthalmitis group and the control group.

Inflammatory markers	Infected endophthalmitis group (54 eyes)	Control group (54 eyes)	*p*-value
WBC × 10^9^/L	9.38 (7.54, 11.62)	7.43 (6.02, 8.93)	0.000^*^
NEUT × 10^9^/L	6.025 (3.81, 9.04)	4.145 (3.03, 5.95)	0.003^*^
LYM × 10^9^/L	2.55 (1.92, 3.14)	2.43 (1.67, 3.19)	0.803
MON × 10^9^/L	0.455 (0.36, 0.69)	0.365 (0.29, 0.45)	0.001^*^
PLT × 10^9^/L	328 (283, 382.5)	313 (241, 380)	0.118
NLR	2.152 (1.10, 4.96)	1.725 (0.99, 3.18)	0.108
MLR	0.177 (0.13, 0.26)	0.159 (0.11, 0.20)	0.032^*^
PLR	130.883 (92.49, 189.83)	115.632 (90.67, 188.23)	0.263
DNLR	0.901 (0.82, 0.93)	0.895 (0.84, 0.93)	0.990
NMLR	2.387 (1.25, 5.44)	1.923 (1.09, 3.35)	0.092
SIRI	1.16 (0.55, 2.12)	0.549 (0.38, 1.15)	0.003^*^
SII	743.922 (302.70, 1634.13)	427.77 (292.39, 1073.20)	0.035*

A comparison between the infectious endophthalmitis group and the control group revealed statistically significant differences in WBC, NEUT, MON, MLR, SIRI, and SII. The following parameters were assessed using the independent samples Mann–Whitney *U*-test: WBC, NEUT, LYM, MON, PLT, NLR, MLR, PLR, dNLR, NMLR, SIRI, and SII (*p* < 0.05).

##### 3.1.3.2 ROC curve analysis of the diagnostic efficacy of each inflammatory index for infectious endophthalmitis after external injury

**Figure 3:**
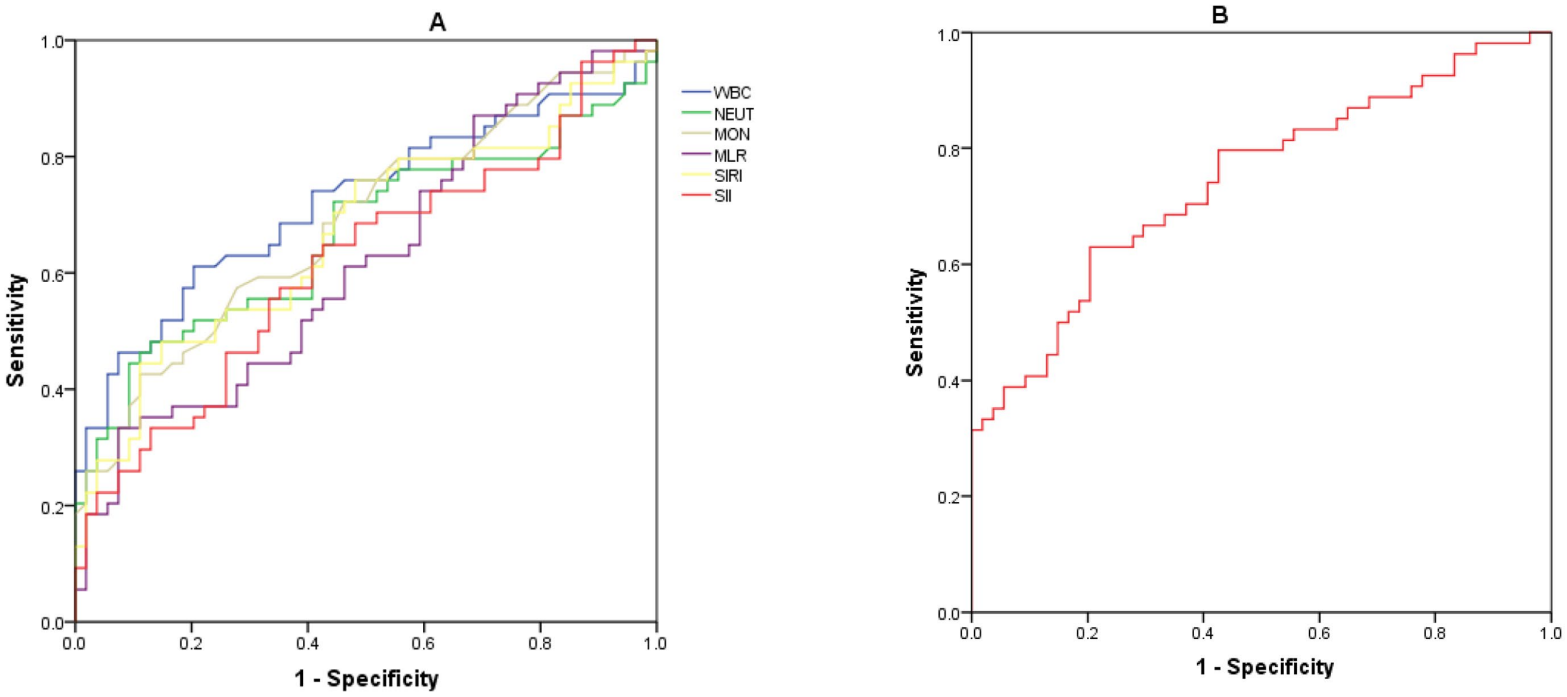
**(A)** The AUC model of inflammatory markers and **(B)** the combined prediction of the above measures resulted in an AUC of 0.745.

The ROC curves of infectious endophthalmitis after external injury were generated for each inflammatory index, and cut-off values, sensitivity, and specificity were calculated, as shown in Table 3 and Figure 3. The AUC of WBC exceeded 0.7 (0.722), indicating good diagnostic efficacy for endopht-halmitis following ocular trauma. The cut-off value for WBC was calculated to be 9.0 × 10^9/L. The SII demonstrated highest sensitivity index (64.8%). MLR showed the highest specificity (92.59%), but its sensitivity was low, suggesting that the negative predictive value of MLR was favorable. Specifically, if the MLR test value in children with ocular trauma was below 0.235, the possibility of endophthalmitis was very low. Combined prediction using the above measures resulted in an AUC of 0.745.

**Table 3:** ROC analysis of inflammatory markers for the diagnosis of post-traumatic infectious endophthalmitis.

Inflammatory markers	AUC	95% CI	Cut-off value	Sensitivity (%)	Specificity (%)
WBC × 10^9^/L	0.722	0.6366–0.817	9	61.1	79.63
NEUT × 10^9^/L	0.665	0.563–0.767	6.265	48.1	87.04
MON × 10^9^/L	0.687	0.587–0.787	0.525	42.6	88.89
MLR	0.620	0.515–0.726	0.235	33.3	92.59
SIRI	0.668	0.566–0.769	1.320	44.4	88.89
SII	0.618	0.512–0.724	510.832	64.8	57.41

For each inflammatory indicator, ROC curves were plotted for post-traumatic infectious endophthalmitis, and the cutoff values, sensitivity, and specificity of each indicator were calculated and displayed.

##### 3.1.3.3 Logistic regression of infectious endophthalmitis factors after trauma

This study further analyzed the risk factors for post-traumatic infectious endophthalmitis, as shown in Table 4. In the univariate logistic regression analysis, time to presentation, WBC, NEUT, MON, MLR, SIRI, and SII were identified as risk factors for poor visual prognosis. Factors with a *p*-value of <0.05 in the univariate regression analysis were included in the multivariate logistic regression models. Time to presentation, SII (with an OR of approximately 1), and NEUT and MON (which were related to MLR and SIRI) were excluded to determine independent predictors of infectious endophthalmitis after trauma in children. WBC count (OR = 1.404) was identified as an independent risk factor in the multivariate logistic regression analysis. These results provide important laboratory evidence of the systemic inflammatory response induced by endophthalmitis and suggest that these indices—particularly WBC—may have important implications for the diagnosis and management of infectious endophthalmitis following trauma in children.

**Table 4:** Univariate and multivariate logistic regression analyses of risk factors associated with poor final visual acuity.

Variables	Beta	SE	Wald	OR (95% CI)	*p*
Univariate analysis					
Rural residence	0.446	0.676	0.435	1.562 (0.415–5.883)	0.509
Time to presentation	0.021	0.006	10.184	1.021 (1.008 to 1.034)	0.001*
WBC	0.346	0.095	13.384	1.413 (1.174 to 1.701)	0.000*
NEUT	0.254	0.08	10.136	1.29 (1.103 to 1.508)	0.001*
MON	3.94	1.277	9.52	51.404 (4.208 to 627.906)	0.002*
MLR	5.115	2.431	4.427	166.58 (1.4193 to 19551.642)	0.035*
SIRI	0.702	0.261	7.244	2.019 (1.21–3.367)	0.007*
SII	0.001	0	5.981	1.001 (1–1.001)	0.014*
Multivariate analysis					
WBC	0.339	0.122	7.796	1.404 (1.106 to 1.782)	0.005*
MLR	2.796	5.128	0.297	16.372 (0.001 to 279261.445)	0.586
SIRI	−0.146	0.458	0.1 02	0.864 (0.352 to 2.121)	0.749

This study further analyzed the risk factors for post-traumatic infectious endophthalmitis.

### 3.2 Prognostic visual acuity factors

#### 3.2.1 Clinical analysis of prognostic visual acuity

Since some younger children were uncooperative during visual acuity testing, 88 patients were ultimately included in the prognostic analysis (68 male patients, 77.27%, 20 female patients, 22.73%). Patients were divided into a favorable outcome group (*n* = 55) and a poor outcome group (*n* = 33). The specific parameters are detailed in Table 5. Within the poor prognosis group, there were three cases of evisceration and four silicone oil-dependent eyes without evidence of apparent phthisis bulbi; one child underwent another surgery due to recurrent retinal detachment.

**Table 5:** Clinical characteristics of the favorable and poor visual outcome groups.

Factor	Favorable prognosis group (55 eyes)	Poor prognosis group (33 eyes)	*p*-value
Endophthalmitis, yes/no	20/35	24/9	0.001*
Eye, Right/Left	25/30	15/18	1.000
Sex, M/F	42/13	26/7	0.793
Family background (rural/urban)	47/8	31/2	0.386
Lens damage, Yes/No	34/21	30/3	0.007*
Gram #, G +/G-	11/2	14/2	1.000
Age	7 (0.82, 0.93)	8 (0.84, 0.93)	0.671
Injury-to-presentation time (hours)	17 (1.25, 5.44)	17 (1.09, 3.35)	0.805
WBC × 10^9^/L	8.05 (0.55, 2.12)	9.82 (0.38, 1.15)	0.015*
NEUT × 10^9^/L	4.75 (302.70, 1634.13)	6.31 (292.39, 1073.20)	0.022*
LYM × 10^9^/L	2.4 (1.68, 3.06)	2.14 (1.65.3.01)	0.515
MON × 10^9^/L	0.43 (0.34, 0.52)	0.42 (0.27, 0.66)	0.590
PLT × 10^9^/L	313 (243, 364)	325 (246, 383)	0.433
NLR	2.093 (0.936, 3.269)	2.939 (1.495, 4.723)	0.092*
MLR	0.173 (0.128, 0.214)	0.181 (0.141, 0.277)	0.289
PLR	118.537 (90.83, 189.130)	158.081 (75.661, 252.905)	0.210
DNLR	0.897 (0.828, 0.930)	0.914 (0.864, 0.951)	0.175
NMLR	2.2 (1.094, 3.454)	3.208 (1.604, 5.015)	0.087*
SIRI	0.785 (0.384, 1.298)	1.22 (0.560, 1.822)	0.053*
SII	472.858 (293.071, 1142.348)	890.187 (290.133, 2205.945)	0.086*

Compared with the poor outcome group, patients with favorable outcomes had a lower proportion of endophthalmitis, less severe lens damage, and significantly lower levels of WBC, NEUT, NLR, NMLR, SIRI, and SII (*p* < 0.05).

#### 3.2.2 Regression analysis and prediction

**Figure 4:**
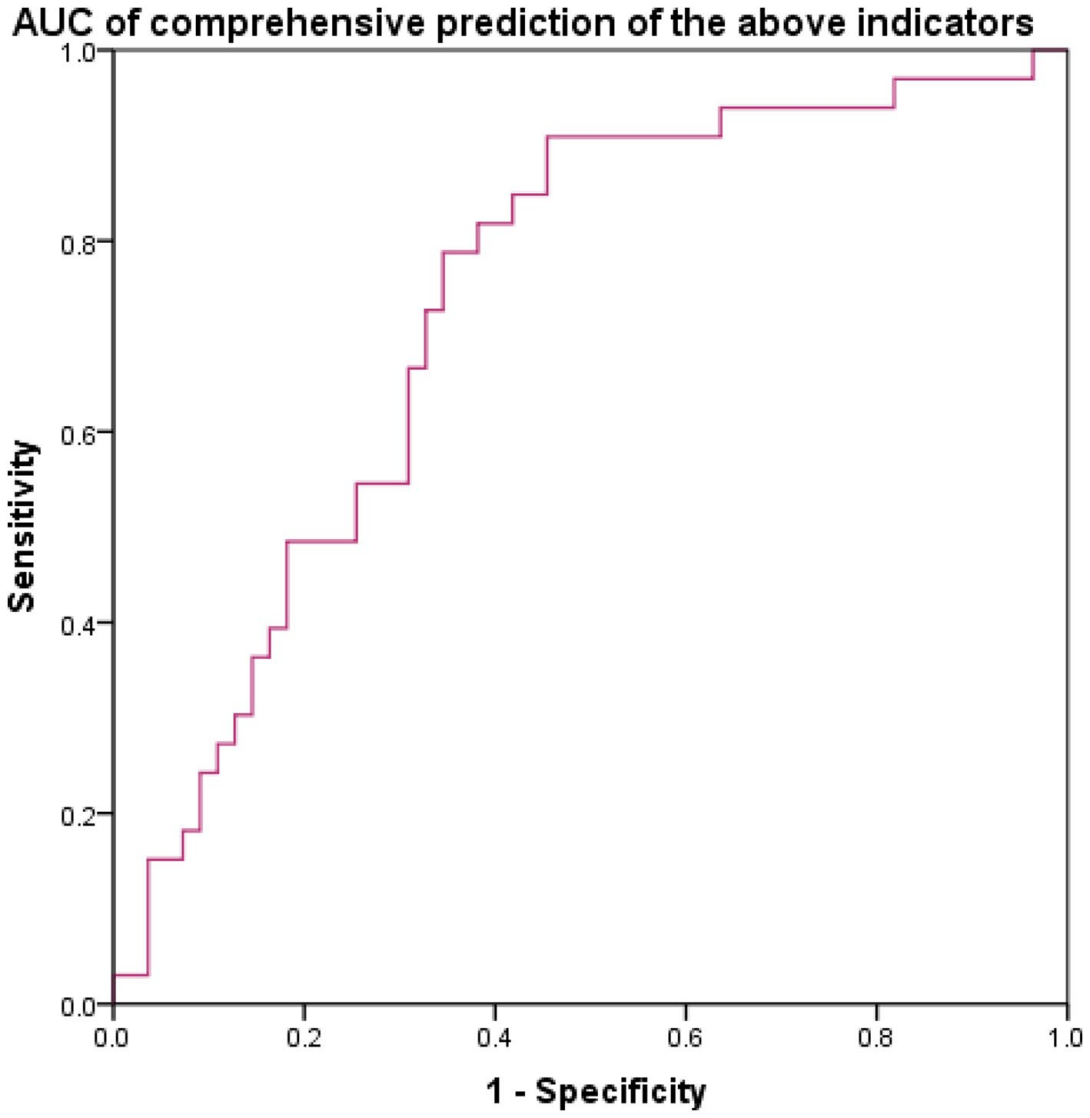
Combined ROC curve analysis of endophthalmitis, lens damage, WBC, and NEUT was performed, AUC = 0.732, sensitivity 90.90%, and specificity 54.55%.

In this study, the risk factors for poor visual prognosis are summarized in Table 6. Parameters, such as endophthalmitis, lens damage, WBC, and NEUT, with a *p*-value of < 0.05 were calculated as significant risk factors for poor visual prognosis. Combined ROC curve analysis of these parameters was performed, AUC = 0.732, sensitivity 90.90%, and specificity 54.55%, as shown in Figure 4. These findings suggest that patients with endophthalmitis, those with severe lens damage, those with high WBC, and those with NEUT are more likely to experience poor vision prognosis.

**Table 6:** Logistic regression analysis of risk factors for poor visual prognosis.

Factor	OR (95% CI)		*p*
Endophthalmitis	4.667 (1.818–11.979)	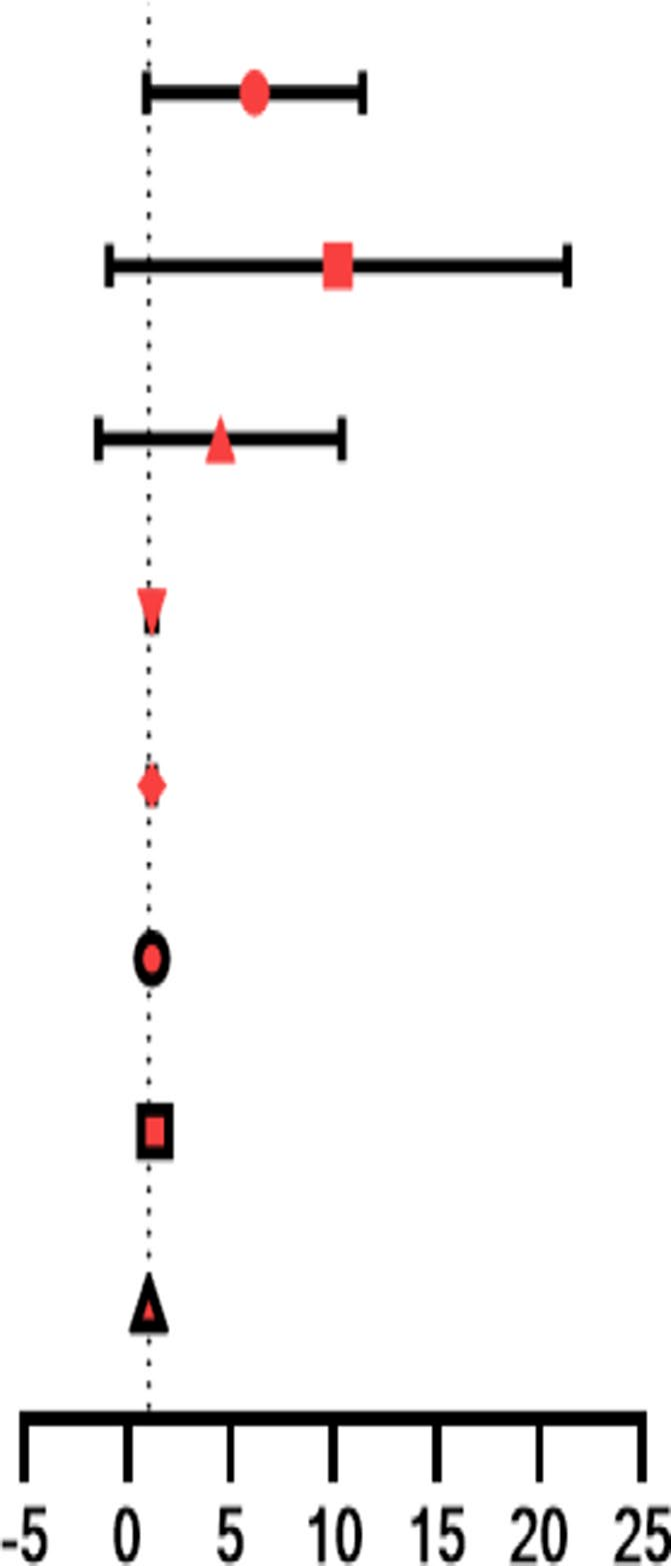	0.001*
Lens Damage	6.176 (1.674 to 22.79)		0.006*
WBC	1.152 (1.001 to 1.326)		0.049*
NEUT	1.15 (1.004 to 1.317)		0.043*
NLR	1.165 (0.988 to 1.375)		0.069
NMLR	1.161 (0.989 to 1.363)		0.067
SIRI	1.26 (0.921 to 1.725)		0.148
SII	1 (1–1.001)		0.058

Endophthalmitis, lens damage, WBC, NEUT were identified as risk factors for poor visual prognosis.

### 3.3 Pathogen culture results

**Figure 5:**
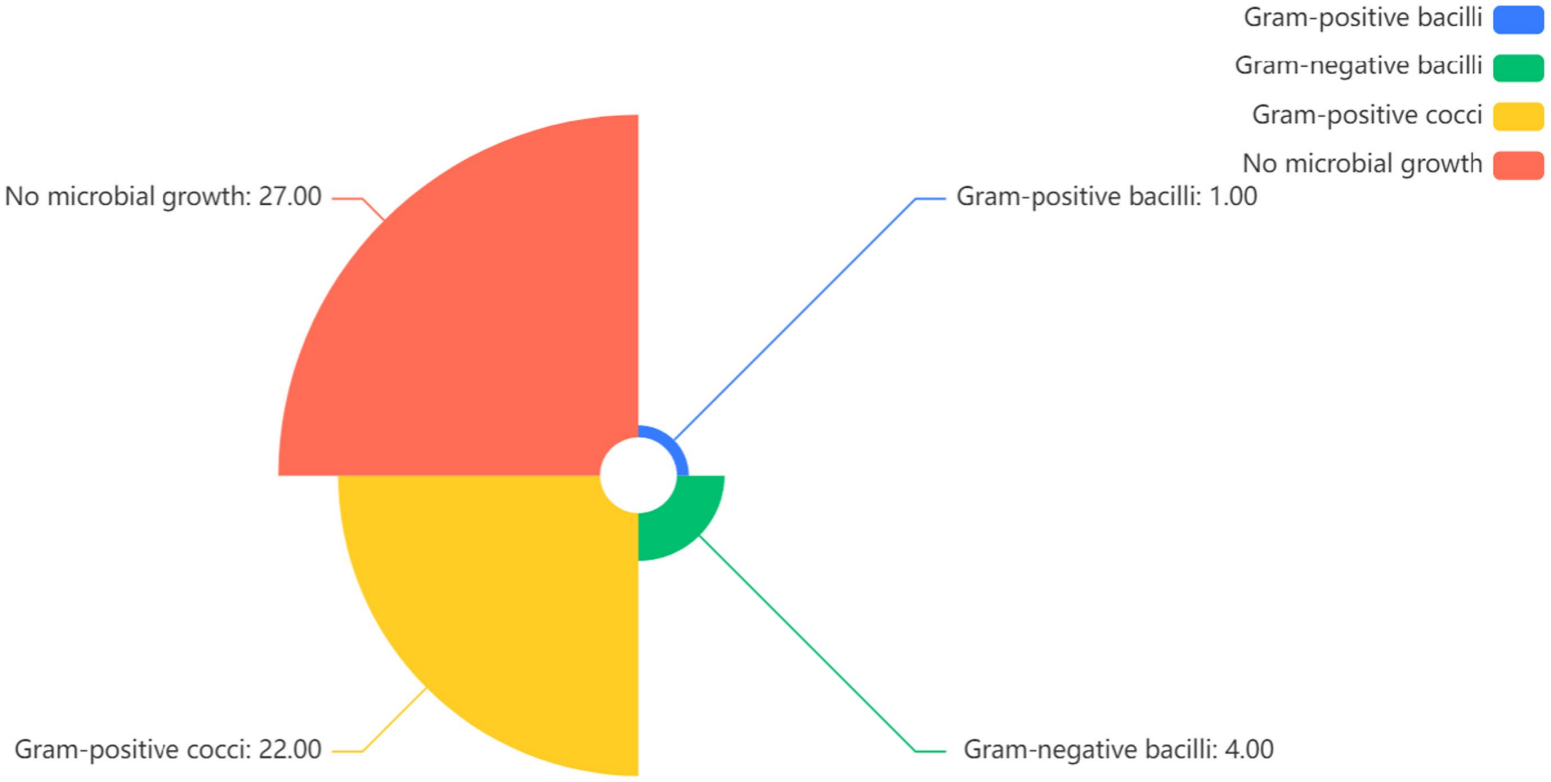
Pathogen analysis of patients with infectious endophthalmitis in children.

Ocular samples were collected from 108 patients for pathogen culture, and a total of 40 samples had positive culture results, with a culture-positive rate of 37.04% (40/108). Bacterial infection was the main type of infection, including Gram-positive cocci in 80% (32/40), Gram-positive bacilli in 7.5% (3/40), and Gram-negative bacilli in 12.5% (5/40), as shown in Figure 5. Among bacterial infections, *Staphylococcus* spp. had the highest detection rate (47.5%, 19/40), of which 10 strains of *Staphylococcus epidermidis* were detected, which accounted for 25% (10/40) of all bacterial isolates. The detection rate of *Streptococcus* was 30% (12/40), which was generally sensitive to cephalosporins, penicillin, vancomycin, aminoglycosides, and quinolones. No fungal infection was identified in the patients.

A total of 27 of 54 endophthalmitis patients had positive culture results, and the positive culture rate was 50% (27/54). Bacterial infection was the main type of infection, including Gram-positive cocci in 81.48% (22/27), Gram-positive bacilli in 3.70% (1/27), and Gram-negative bacilli in 14.81% (4/27), as shown in Figure 5. Among the bacterial infections, *Staphylococcus* and *Streptococcus* had the highest detection rate (37.04%, 10/27) and were generally sensitive to cephalosporins, penicillin, vancomycin, aminoglycosides, and quinolones.

## 4 Discussion

The development of traumatic endophthalmitis in children is closely associated with behavioral characteristics, weak safety awareness, and thinner ocular walls. Since ocular structures in children are not fully developed and their immune systems are relatively immature, infections often progress rapidly and are associated with poor outcomes (6, 7). However, given the relatively low incidence of this condition in the pediatric population, related research remains limited. This study aimed to provide a comprehensive analysis of the clinical characteristics, microbiological spectrum, inflammatory markers, and prognostic factors associated with pediatric post-traumatic infectious endophthalmitis, thereby offering evidence-based insights for clinical diagnosis and management.

The epidemiological profile of pediatric ocular trauma shows a clear sex disparity, with boys being more frequently affected than girls. Previous studies have suggested that this phenomenon may be related to boys’ behavioral patterns, including greater engagement in high-risk activities, more frequent use of sharp objects, and increased participation in outdoor play (9–11). A higher proportion of cases occurred in rural children, likely due to lower educational attainment and weaker awareness of safety precautions. These findings highlight the urgent need for targeted safety education in this vulnerable group.

In this study, comparison between groups revealed that children with post-traumatic infectious endophthalmitis were more often from rural areas, presented later after injury, and had poorer post-treatment visual outcomes than controls. This finding suggests that the injuries sustained in the endophthalmitis group were more severe, with treatment delays corresponding disease progression. Consistent with the results of this study, Bohrani Sefidan et al. (11) reported metallic objects as the most common cause of pediatric ocular trauma, while other studies have identified plant-related injuries as the predominant etiology (12). The findings of this study further showed that metallic injuries were significantly more common in the endophthalmitis group, whereas glass/ceramic-related injuries were more prevalent in the control group. This may be explained by the high contamination potential of metallic objects (particularly with Gram-positive bacteria), their high kinetic energy and strong penetrating capacity, the irregular and fragmented nature of injuries they produce, and their propensity to promote bacterial biofilm formation. In contrast, glass and ceramic objects, though also common causes of injury, generally carry a lower microbial load, weaker virulence, and produce comparatively “clean” wounds with milder intraocular reactions, explaining their higher prevalence in the control group.

Among the 13 patients in the control group, positive bacterial culture results were observed. This finding may be attributed to several factors. First, in 10 cases where the anterior chamber had collapsed, rendering aqueous humor sampling unfeasible, conjunctival sac secretions were collected for culture. Additionally, in 3 patients, samples were obtained from prolapsed vitreous or iris surface secretions, which carried a potential risk of conjunctival sac contamination. Second, all detected bacteria—including *Staphylococcus epidermidis*, *Streptococcus*, *Staphylococcus aureus*, *Acinetobacter haemolyticus*, and *Corynebacterium pseudodiphtheriticum*—represent species commonly present as normal conjunctival flora (13, 14). Therefore, the isolation of these microorganisms from conjunctival secretions does not necessarily indicate true infection. Finally, all patients received medical attention within 24 h post-surgery and were promptly treated with antibiotics. The efficacy of the antimicrobial therapy exceeded the virulence of the detected bacteria, thereby preventing the development of intraocular infection.

Inflammatory markers including WBC, NEUT, MON, MLR, SIRI, and SII were significantly elevated in pediatric post-traumatic endophthalmitis, indicating higher disease risk and reflecting both infection severity and systemic inflammatory response. Elevated WBC reflects infection severity and systemic inflammation (15), while increased neutrophils and monocytes suggest active innate immune engagement (16), while the composite indices (MLR, SIRI, SII) provide a more comprehensive assessment of systemic inflammation than single parameters, supporting their utility as practical biomarkers for evaluating severity and inflammatory status in this condition. These findings align with previous studies supporting the value of these composite indices such as MLR, SIRI, and SII in inflammatory assessment (17–20). Elevated MLR, SIRI, and SII may indicate heightened neutrophil and monocyte activity, providing useful biomarkers for evaluating systemic inflammation and severity in endophthalmitis.

Delayed medical presentation was also confirmed as a critical risk factor for pediatric post-traumatic endophthalmitis. Delay in primary wound repair has long been recognized as one of the most important risk factors for endophthalmitis after ocular trauma (6, 21). The American Eye Injury Registry recommends primary repair of open-globe injuries within 24 h (22). However, in young children, the inability to articulate symptoms often results in delays in seeking care, thereby increasing the risk of infection. Narang et al. (23) reported that 48.62% of pediatric patients presented after 24 h, and wound repair delayed beyond 72 h was significantly associated with endophthalmitis. Thompson et al. (24) similarly found that delays exceeding 24 h increased the risk fourfold. Zhang et al. (21) further demonstrated that initial repair within 24 h had a protective effect against the development of endophthalmitis. Reported reasons for delayed presentation include long distance to medical facilities (40.5%), financial constraints (22%), neglect (19.7%), delayed referral (10.6%), and absence of symptoms (9.1%) (24).

Lens injury was identified in our study as an independent risk factor for poor visual outcome, consistent with prior research, which has established lens rupture as a strong predictor of post-traumatic suppurative endophthalmitis (10, 25–27). Mechanistically, lens damage compromises the refractive medium and visual axis, while rupture exposes lens proteins, triggering severe phacoanaphylactic uveitis. Moreover, the disrupted lens provides a favorable medium for bacterial proliferation, substantially increasing the risk of infection. For these reasons, thorough removal of the injured lens and capsule is regarded as a critical therapeutic principle to eradicate infectious foci and inflammatory stimuli (28).

Our study also confirmed the distinctive microbiological profile of pediatric infectious endophthalmitis. Consistent with previous reports, Gram-positive cocci were the most common pathogens, with *Streptococcus* and *Staphylococcus* species predominating (9, 29). Chinese studies have similarly shown that Gram-positive cocci account for over 60% of isolates, with *Streptococcus pneumoniae*, *Streptococcus pyogenes*, and *Staphylococcus aureus* being the most common pathogens. Yang et al. (9) specifically noted that *β*-hemolytic *Streptococcus* was the leading pathogen in preschool children, characterized by strong virulence and the ability to rapidly cause severe intraocular infection. Although Gram-positive organisms predominate, Gram-negative bacteria also play an important role. Li et al. (30) and Tang et al. (31) reported that *Escherichia coli*, *Pseudomonas aeruginosa*, and *Klebsiella pneumoniae* accounted for approximately 10.7% of isolates in traumatic endophthalmitis. In particular, *P. aeruginosa* is highly destructive due to the production of proteases and other virulence factors, causing profound intraocular tissue damage within a short time (31).

This study has several limitations. First, the relatively low incidence of pediatric infectious endophthalmitis limited our sample size. Second, as a retrospective, single-center study, it may be subject to selection and information biases. Third, regional and ethnic variations may limit the generalizability of these findings. Large-scale, multicenter epidemiological studies are warranted to better elucidate the incidence, risk factors, and pathogen spectrum of pediatric endophthalmitis and to guide evidence-based preventive strategies.

In conclusion, pediatric ocular trauma predominantly affects boys and rural children. WBC count showed good diagnostic efficiency for post-traumatic infectious endophthalmitis, and combined predictive models further improved diagnostic accuracy. Delayed presentation was a key risk factor for infection. Endophthalmitis, lens injury, elevated WBC, and elevated NEUT were identified as risk factors for poor visual prognosis. Bacterial infections predominated, with *Streptococcus* and *Staphylococcus* species being the leading pathogens. Upon patient admission, the timing for sample collection should be promptly evaluated, and samples should be obtained as early as possible. When there is high clinical suspicion of endophthalmitis or in cases of full-thickness scleral wounds, vitreous samples should be collected and sent for analysis. WBC count exceeding 9.0 × 10^9/L should alert clinicians to the potential development of endophthalmitis.

## Data Availability

The original contributions presented in the study are included in the article/supplementary material, further inquiries can be directed to the corresponding author.
